# Targeting of RUNX3 by miR-130a and miR-495 cooperatively increases cell proliferation and tumor angiogenesis in gastric cancer cells

**DOI:** 10.18632/oncotarget.5037

**Published:** 2015-09-07

**Authors:** Sun Hee Lee, Yuk Dong Jung, Young Sun Choi, You Mie Lee

**Affiliations:** ^1^ Research Institute of Pharmaceutical Sciences, College of Pharmacy, Daegu, 702-701, Republic of Korea

**Keywords:** cell proliferation, miR-130a, miR-495, RUNX3, angiogenesis

## Abstract

Mature microRNAs (miRNAs) are 21 to 23 nucleotide noncoding RNA molecules that can downregulate multiple gene expression by mRNA degradation or translational repression. miRNAs are considered to play important roles in cell proliferation, apoptosis, and differentiation during mammalian development. The Runt-related transcription factor 3 (RUNX3) expression and activity are frequently downregulated by various mechanisms in gastric cancer. We have reported that RUNX3 inactivation is crucial for early tumorigenesis. In this study, we investigated the role of miRNAs targeting RUNX3 in early tumorigenesis. miR-130a and miR-495 upregulated under hypoxic conditions that bind to the RUNX3 3′-untranslated region (3′-UTR) were identified in gastric cancer cells by using microarray analysis and bioinformatics programs. Combination of miR-130a and miR-495 inhibited RUNX3 expression at the protein level, but not at the mRNA level. miR-130a and miR-495 significantly inhibited the RUNX3–3′UTR-luciferase activity. Combination of miR-130a and miR-495 significantly decreased apoptosis determined by Annexin V-FITC/propidium iodide staining and flow cytometric analysis, and the expression of Bim in SNU484 gastric cancer cells. In addition, p21 and Bim, RUNX3 target genes, were completely downregulated by the combination of miR-130a and miR-495. Using matrigel plug assay, we found that antagomiRs specific for miR-130a and miR-495 significantly reduced angiogenesis *in vivo*. In conclusion, targeting miR-130a and miR-495 could be a potential therapeutics to recover RUNX3 expression under hypoxic conditions and in early tumorigenic progression.

## INTRODUCTION

MicroRNAs (miRNAs) are 21–23 nucleotide RNA molecules that modulate the stability or translational efficiency of target messenger RNAs [1, 2]. The biogenesis of miRNAs begins with a primary transcript, termed the pri-miRNA, and the combined action of Drosha and Dicer ribonucleases generates the mature miRNA species [3]. miRNAs binding to 3′-untranslated region (UTR) of the targeted mRNA leads to its degradation or translational repression [4]. miRNAs regulate a variety of biological processes, including cellular differentiation, proliferation, metabolic signaling, and apoptosis [4]. Differential expression of miRNAs between normal and tumor tissues has been observed in various cancer types, suggesting a possible link between miRNA expression and the development of cancer [5]. Deregulated miRNAs may function as tumor suppressors such as miR-205 and let-7 [6, 7], or oncogenes such as miR-17–92 cluster and miR-214 [8, 9], depending on the regulated targets. Therefore, we proposed that deregulation of candidate miRNAs could be a possible mechanism for the downregulation of tumor suppressors in tumors.

The Runt family of transcription factors consists of three members, RUNX1, RUNX2, and RUNX3. All three RUNXs play important roles in both the normal developmental process and carcinogenesis [10]. RUNX3 is related to neurogenesis of the dorsal root ganglia, T-cell differentiation, and tumorigenesis of the gastric epithelium. Primary gastric cancer specimens express lower levels of RUNX3 due to a combination of a hemizygous deletion and hypermethylation of the RUNX3 promoter region [10]. The gastric epithelium of *Runx3* knockout mice exhibits hyperplasia, a reduced rate of apoptosis, and reduced sensitivity to TGF-β, suggesting that RUNX3 tumor suppressor operates downstream of the TGF-β signaling pathway. TGF-β receptors and their downstream signal transducers, SMADs, are frequently inactivated in various cancers [11]. RUNX3 cooperates with SMAD3/4 to activate TGF-β-dependent growth inhibition and apoptosis by induction of p21 and Bim, respectively [12, 13]. Functional inactivation of RUNX3, through hypermethylation of its promoter region, hemizygous deletion, epigenetic silencing, or cytoplasmic mislocalization, is frequently observed in solid tumors of diverse origins. *Runx3* targeted deletion in mouse lung resulted in lung adenomas and abrogated the cellular defense mechanism against oncogenic activation, suggesting that *Runx3* plays critical roles in normal differentiation and suppression of tumor initiation [14]. Hypoxia, often found in solid tumors larger than 1 mm^3^, as well as in pathophysiolocial premalignant conditions [15], downregulates RUNX3 by promoter histone deacetylation and methylation in gastric cancer cells [16], suggesting that histone modification plays a role in RUNX3 inactivation in early tumorigenesis and tumor growth. Moreover, hypoxia-inducible factor-1α (HIF-1α), a key transcription factor that induces angiogenesis and tumor aggressiveness is destabilized by RUNX3 [17]. Together, these data indicate that RUNX3 inactivation is a major risk factor in early tumorigenesis and its inactivation contributes to tumor progression via increasing angiogenesis [10, 17–20]. In this study, we examined the combination effect of miRNA-130a and miRNA-495 targeting RUNX3 under hypoxic conditions in cell proliferation and angiogenesis in gastric cancer cells.

## RESULTS

### Identification of miRNAs that bind to RUNX3 3′-UTR, using microarray data and bioinformatics

**Figure 1:**
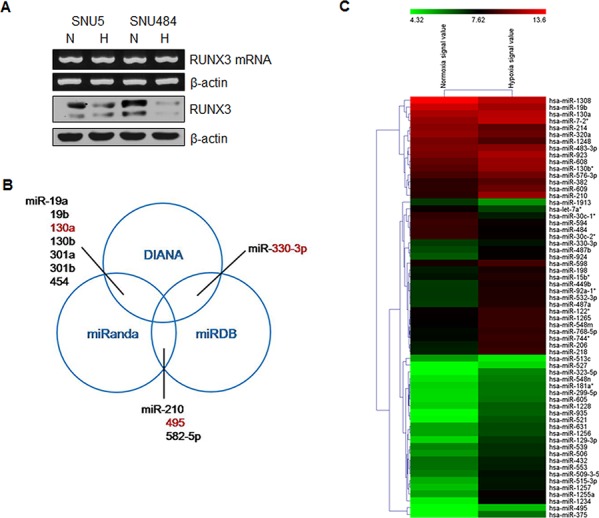
Identification of miRNAs targeting RUNX3 under hypoxic conditions **A.** SNU5 and SNU484 cells were incubated under normoxia (N) or hypoxia (H) for 24 h. RUNX3 expression patterns were examined by semiquantitative RT–PCR and western blot analysis in gastric cancer cells. β-actin expression served as an internal control. **B.** Venn diagram shows potential miRNAs targeting RUNX3 mRNA 3′-UTR, predicted by three algorithms, DIANA, miRanda, and miRDB. **C.** The expression of miRNAs was analyzed using total RNA from gastric cancer cells exposed to hypoxia for 24 h. Heatmap demonstrates the differential expression pattern of miRNAs between normoxia and hypoxia.

We previously reported that hypoxia decreased *RUNX3* mRNA expression by histone modifications [16]. However, in specific gastric cancer cells such as SNU5 and SNU484 cells, *RUNX3* mRNA expression was not decreased, but its protein level was reduced under hypoxic conditions (Figure 1A). We then focused on regulation mechanism of RUNX3 protein expression by miRNAs at the post-transcriptional level. To identify possible miRNAs negatively regulating RUNX3 protein expression, we performed a bioinformatics analysis using the three algorithmic databases (DIANA, miRanda, and miRDB). We identified 11 potential miRNAs targeting RUNX3 mRNA 3′-UTR from these databases (Figure 1B). Moreover, we performed miRNA microarray analysis to identify hypoxia-induced miRNAs. After exposure of gastric cancer cells to hypoxia, the expression levels of a number of miRNAs were significantly changed (Figure 1C). Among the upregulated miRNAs under hypoxic conditions, we selected three miRNAs, miR-130a, miR-330-3p, and miR-495, which overlapped with the 11 miRNAs targeting RUNX3, from multiple algorithmic databases.

### miR-130a and miR-495 downregulate endogenous RUNX3 expression

**Figure 2:**
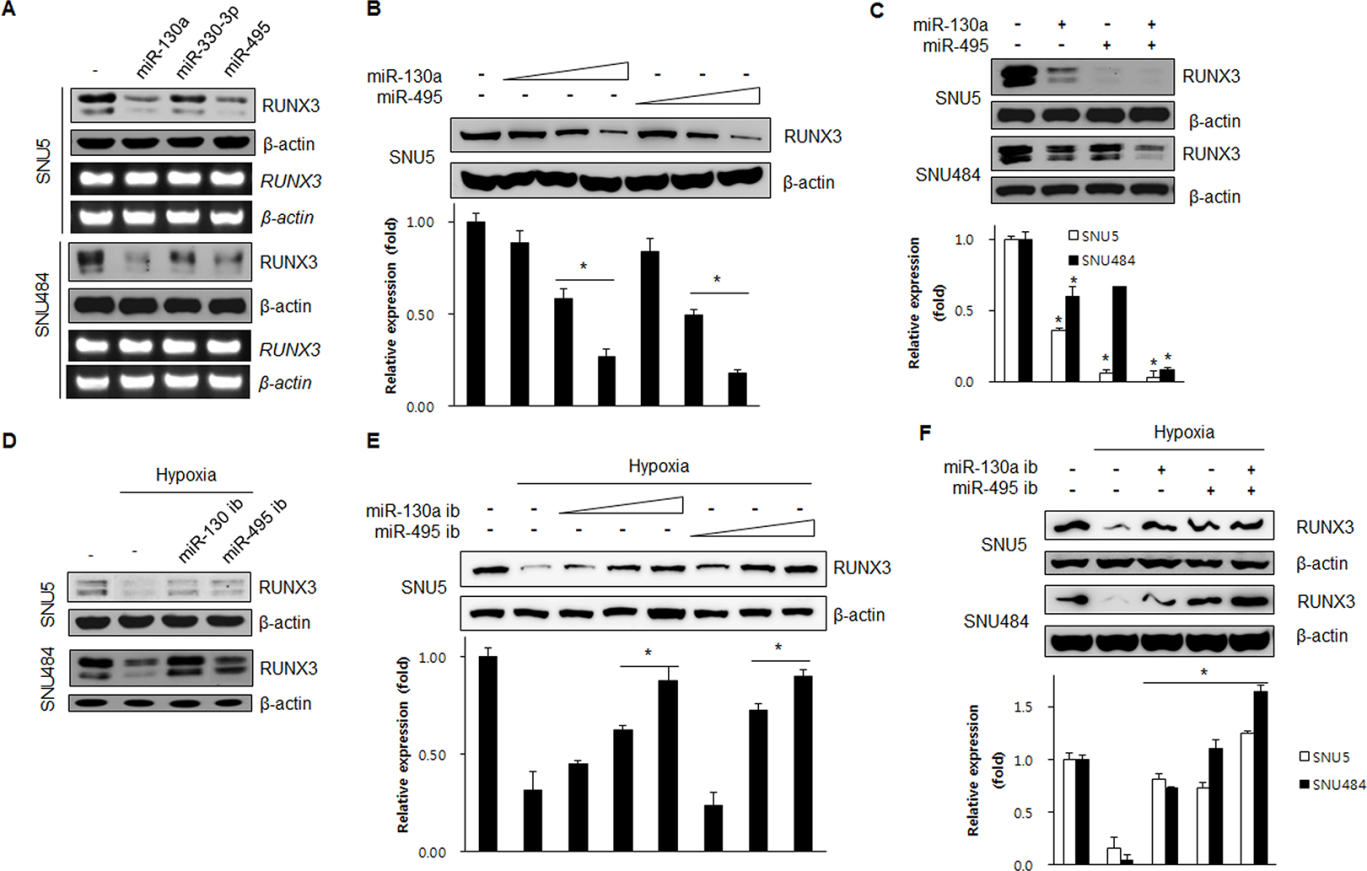
miR-130a and miR-495 inhibit RUNX3 protein expression **A.** SNU5 and SNU484 cells were harvested 24 h after transfection with miRNA mimics (10 nM). The resulting changes in RUNX3 expression levels were examined by western blot and RT-PCR. **B.** SNU5 cells transfected with various doses of miR-130a and miR-495 mimics (1, 5, and 20 nM). RUNX3 protein expression was analyzed by western blot. *p < 0.05 *vs.* between indicated two groups. **C.** SNU5 and SNU484 cells transfected with the miRNA mimics (10 nM) individually or in combination. The resulting changes in RUNX3 expression levels were examined by western blot and RT-PCR (*n* = 3). *p < 0.05 *vs*. negative control. **D.** SNU5 and SNU484 cells were harvested 24 h under normoxia or hypoxia after transfection with miRNA inhibitors (50 nM). The cell lysates were subjected to western blot (*n* = 3). **E.** SNU5 cells transfected with various doses of miR-130a and miR-495 inhibitors (10, 50, and 70 nM) and exposed to normoxia or hypoxia for 24 h. RUNX3 protein expression was analyzed by western blot (*n* = 3). *p < 0.05 between indicated two groups. **F.** SNU5 and SNU484 cells transfected with the miRNA inhibitors (50 nM) individually or in combination under normoxia or hypoxia for 24 h. Cell lysates were subjected to western blot (*n* = 3). *p < 0.05 *vs*. hypoxic control.

To determine how miR-130a, miR-330-3p, and miR-495 regulate RUNX3 expression, we transfected the mimics of miR-130a, miR-330-3p, and miR-495 and checked RUNX3 protein and mRNA expression by western blot and RT-PCR, respectively. As shown in Figure 2A, miR-130a and miR-495 reduced the expression of endogenous RUNX3 protein, but not the mRNA level, indicating that miR-130a and miR-495 negatively regulate RUNX3 protein expression. However, miR-330-3p did not affect RUNX3 protein expression. Thus, we further investigated the role of miR-130a and miR-495 in the regulation of RUNX3 expression. Increasing concentrations of either miR-130a or miR-495 reduced RUNX3 protein expression (Figure 2B). Next, when cells were transfected with miR-130a and miR-495 mimics, either individually or in combination, these two miRNAs significantly reduced RUNX3 expression in a cooperative manner (Figure 2C). Transfection of antisense miR-130a and antisense miR-495 (inhibitors, ib) allowed the recovery of endogenous RUNX3 protein expression (Figure 2D). Increasing the dose of miRNAs inhibitors induced RUNX3 protein expression in a dose-dependent manner (Figure 2E). Recovery of RUNX3 expression was more prominent when using a combination of these two miRNA inhibitors at low dose in a cooperative manner (Figure 2F).

These results suggested that miR-130a and miR-495 downregulate RUNX3 protein expression at the post-transcriptional level under hypoxic conditions.

### miR-130a and miR-495 are upregulated under hypoxic conditions in SNU5 and SNU484, but not in MKN45

**Figure 3:**
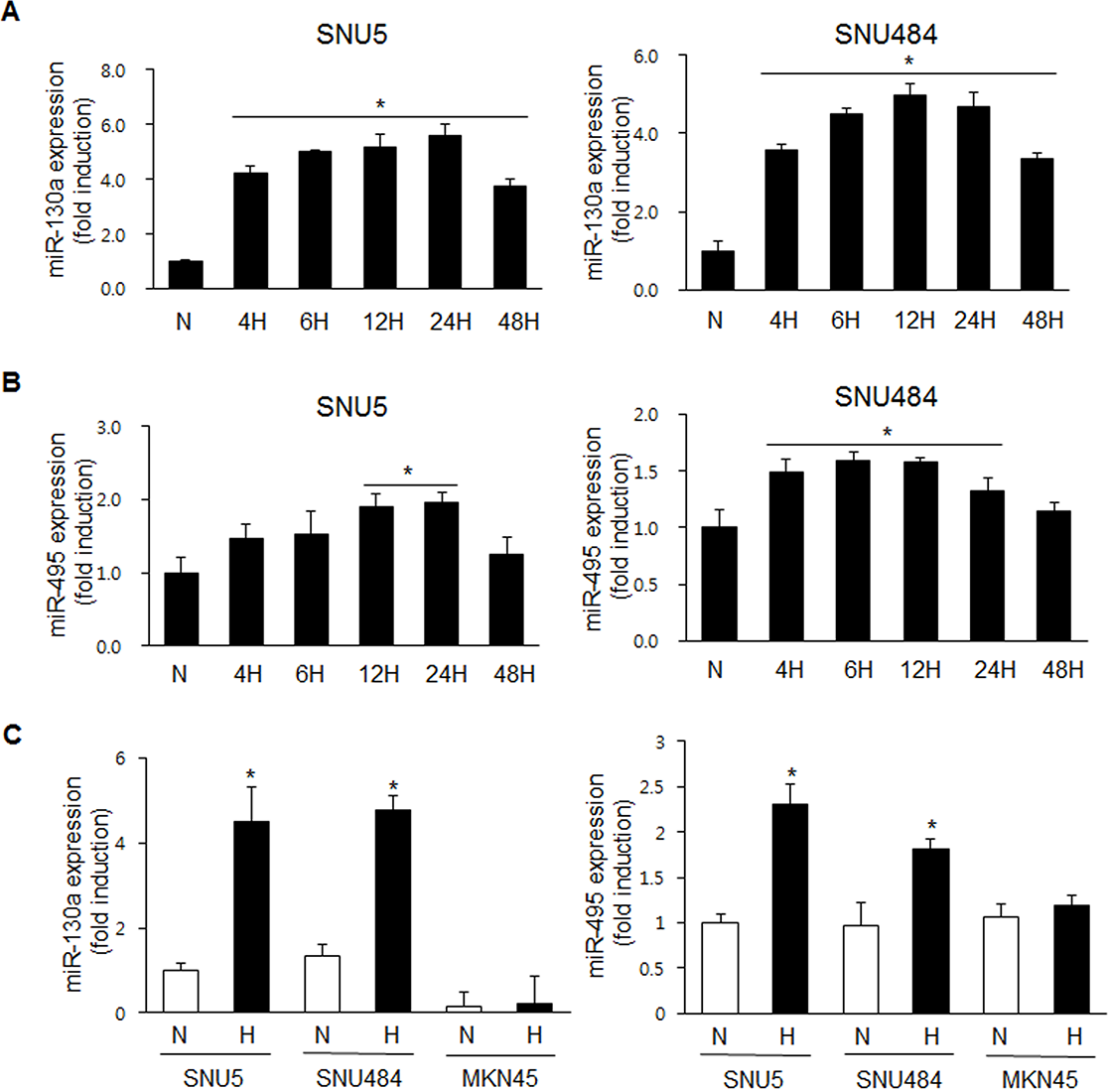
miR-130a and miR-495 are induced by hypoxic conditions in SNU5 and SNU484 cells **A.** SNU5 and SNU484 cells were exposed to hypoxic conditions for the indicated time. Mature miR-130a expression was detected by quantitative RT-PCR (*n* ≥ 3, triplicate). *p < 0.01 *vs*. N (normoxic) control. **B.** SNU5 and SNU484 cells were exposed to hypoxic conditions for the indicated time. Mature miR-495 expression was detected by quantitative RT-PCR (*n* ≥ 3, triplicate). *p < 0.05 *vs*. N (normoxic) control. **C.** SNU5, SNU484, and MKN45 cells were exposed to hypoxia. Mature miR-130a and miR-495 expression was detected by miRNA qRT-PCR (*n* ≥ 3, triplicate). *p < 0.05, **p < 0.01 *vs*. N (normoxic) control.

To investigate whether hypoxia regulates miR-130a and miR-495 expression in SNU5 and SNU484 cells, SNU5 and SNU484 cells were exposed to hypoxia at the indicated time points. As expected, miR-130a and miR-495 levels were strongly increased by real-time PCR under hypoxic conditions (Figure 3A and 3B). Because our previous report demonstrates that *RUNX3* mRNA level is downregulated in MKN45 cells, but its protein level is downregulated in SNU5 cells under hypoxic conditions [16], we checked the expression levels of miR-130a and miR-495 in MKN45 and SNU5 cells. miR-130a and miR-495 expression was strongly increased in SNU5 and SNU484, but not in MKN45 cells under hypoxia (Figure 3C). These results suggest that RUNX3 protein was downregulated by miR-130a and miR-495 under hypoxic conditions in SNU5 and SNU484.

### RUNX3 is a direct target gene of miR-130a and miR-495

**Figure 4:**
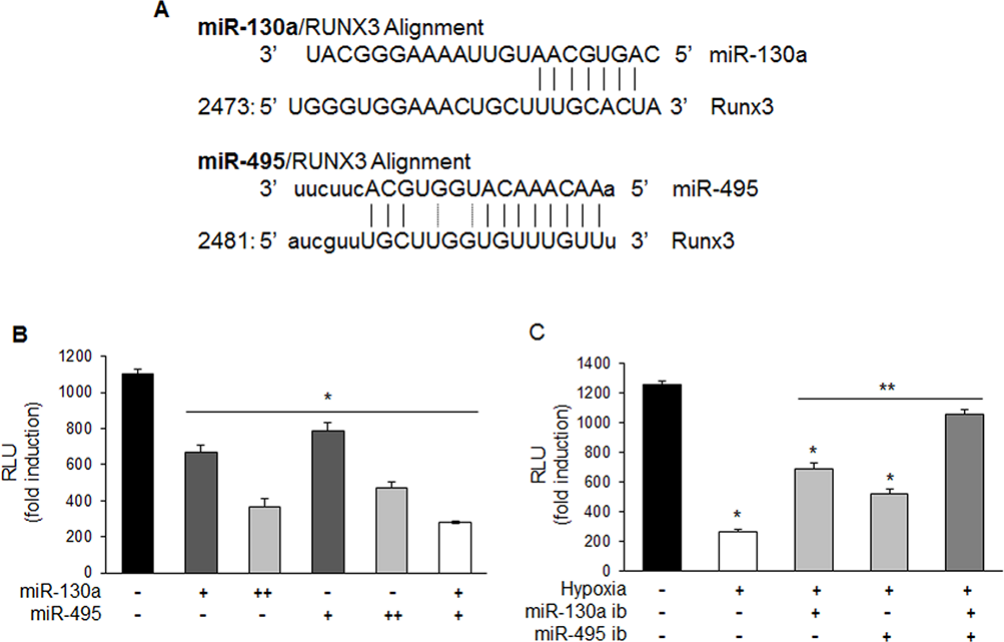
miR-130a and miR-495 directly target 3′-UTR of RUNX3 mRNA **A.** Schematic representation of miR-130a and miR-495 target binding site in the 3′-UTR of *RUNX3* mRNA. **B.** MKN1 cells were transfected with pRUNX3 3′-UTR with miR-130a and miR-495 mimics (10 and 50 nM) and luciferase activity was determined. Relative luciferase activity was determined by normalization to β-galactosidase activity (*n* ≥ 3, triplicate). *p < 0.05 *vs*. control. **C.** MKN1 cells were transfected with pRUNX3 3′-UTR with miR-130a and miR-495 inhibitors (50 nM) and exposed to hypoxia. Luciferase activity was determined. Relative luciferase activity was determined by normalization to β-galactosidase activity (*n* ≥ 3, triplicate). *p < 0.01 *vs*. negative control, **p < 0.01 *vs*. hypoxic control.

To investigate whether miR-130a and miR-495 could bind directly to the 3′-UTR of *RUNX3* mRNA to decrease RUNX3 expression, we cloned these miRNAs target site in the 3′-UTR of *RUNX3* into the luciferase expressing pGL3-control vector just downstream of the luciferase open reading frame (Figure 4A). Cells were transfected with pGL3-RUNX3 3′-UTR reporter and various doses of miR-130a and miR-495 mimics either individually or in combinations. The luciferase activity of RUNX3 3′-UTR was decreased in the presence of miR-130a and miR-495 mimics compared to the control. Combination of miR-130a and miR-495 synergistically downregulated RUNX3 reporter activity (Figure 4B). As shown in Figure 4C, luciferase activity of RUNX3 3′-UTR was recovered when cells were transfected with the pRUNX3 3′-UTR reporter and miR-130a and miR-495 inhibitors either individually or in combination (Figure 4C). These results indicate that miR-130a and miR-495 impair *RUNX3* mRNA translation by directly binding to the 3′-UTR of RUNX3.

### miR-130a and miR-495 promote cell proliferation and tumor angiogenesis of gastric cancer cells

**Figure 5:**
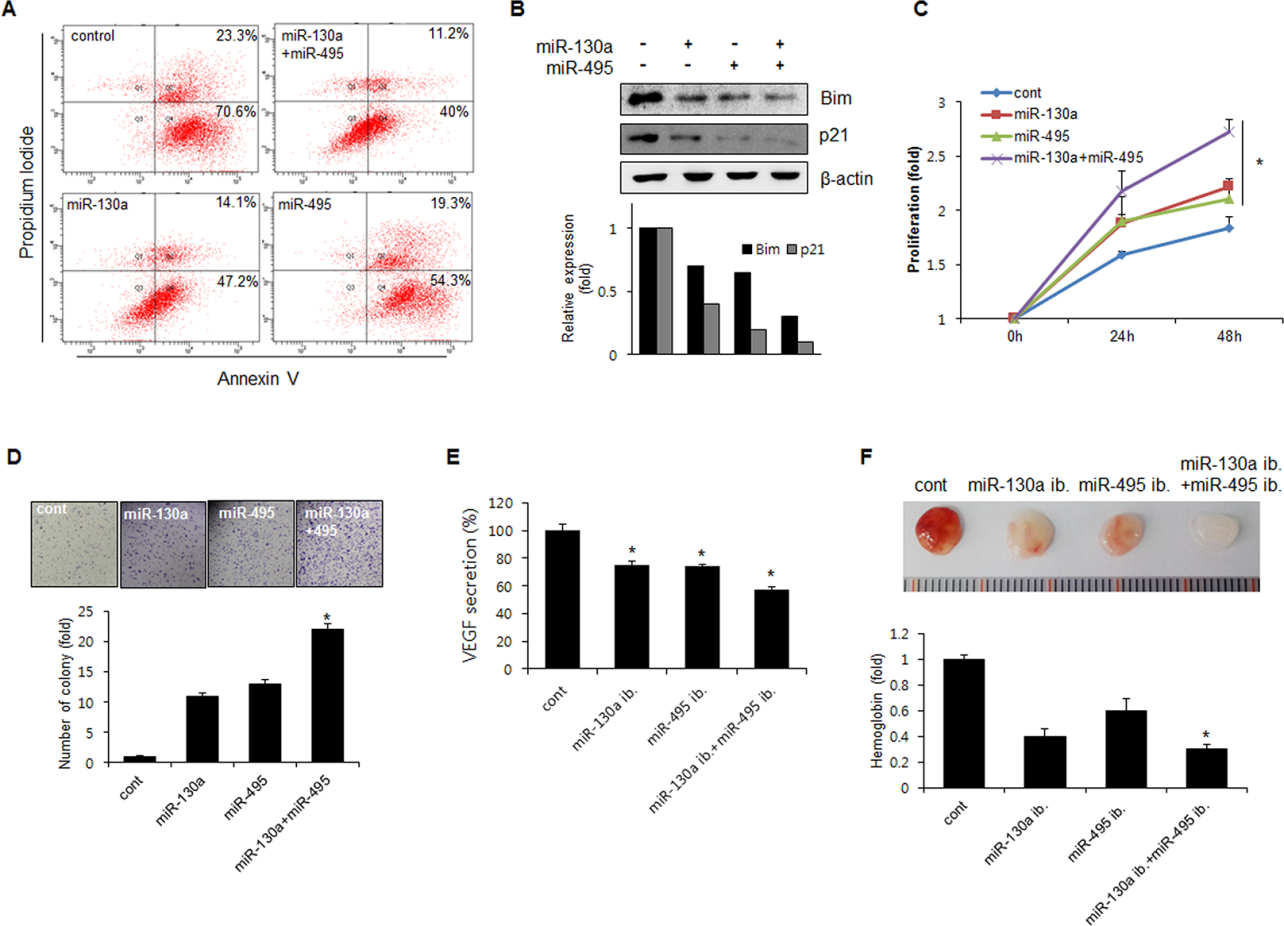
miR-130a and miR-495 increases cell proliferation and tumor angiogenesis **A.** SNU484 cells were transfected with miR-130a and miR-495 mimics (10 nM). Apoptotic cells were monitored by annexin V-FITC/PI staining and flow-cytometry analysis. The right-lower quadrant shows early apoptotic cells, while the right-upper quadrant shows late apoptotic cells. **B.** SNU484 cells were transfected with miR-130a and miR-495 mimics (10 nM). Expression of p21 and Bim was assessed by western blot. **C.** BrdU incorporation assays were performed 24 and 48 h after the transfection of SNU484 cells with miR-130a and miR-495 mimics (20 nM) (*n* ≥ 3, triplicate). *p < 0.01 *vs*. control. **D.** The cell growth capacity of SNU484 cells transfected with miR-130a and miR-495 mimics (20 nM) was detected by colony formation assays (*n* ≥ 3, triplicate). *p < 0.05. **p < 0.01 *vs*. control. **E.** Conditioned medium of SNU484 cells transfected with miR-130a and miR-495 inhibitors (50 nM) were collected and levels of secreted VEGF were determined by ELISA. **F.** Matrigel was presoaked in conditioned medium of SNU484 cells transfected with miR-130a and miR-495 inhibitors (50 nM) and injected subcutaneously into C57BL/6 mice. After 7 days, the matrigel plugs were removed and photographed (*n* ≥ 5, triplicate). *p < 0.05. **p < 0.01 *vs*. control.

RUNX3 is a tumor suppressor in gastric cancer and inactivation of RUNX3 is causally associated with human gastric carcinogenesis. p21 and Bim are direct downstream targets of RUNX3 that activate RUNX3-dependent apoptosis and inhibition of proliferation. To investigate the role of miR-130a and miR-495 in the function of gastric cancer cells, we determined the synergistic effect of miR-130a and miR-495 on apoptosis. The extent of gastric cancer cell apoptosis was monitored by flow cytometry after annexin V/PI staining. The combination of miR-130a and miR-495 significantly reduced the percentage of apoptotic cells (Figure 5A). Next, we analyzed the expression of RUNX3 target molecules, p21 and Bim. Figure 5B shows that, compared with control cells, cells transfected with a combination of miR-130a and miR-495 showed a marked decrease in p21 and Bim expression. To determine the role of miR-130a and miR-495 in the proliferation of gastric cancer cells, SNU484 cells were transfected with miR-130a and miR-495 mimics, and cell survival was assessed. miR-130a and miR-495 significantly increased cancer cell proliferation (Figure 5C). The anchorage-independent colony formation ability was increased by approximately 50% in SNU484 cells transfected with miR-130a and miR-495 mimics compared with that of control cells (Figure 5D). These data indicated that the combination of miR-130a and miR-495 effectively induced the proliferation of gastric cancer cells.

Next, we tested the involvement of miR-130a and miR-495 in angiogenesis. VEGF secretion into media was significantly decreased by miR-130a and miR-495. Combination of miR-130a and miR-495 potently inhibited VEGF secretion (Figure 5E). To confirm this result *in vivo*, mice were subcutaneously injected with matrigel plugs presoaked in conditioned medium obtained from miR-130a inhibitor- and miR-495 inhibitor-transfected cancer cells. As expected, the angiogenic activity induced by miR-130a and miR-495 inhibitors was lower than that of control (Figure 5F). These data indicated that antagomiRs specific for miR-130a and miR-495 significantly reduced angiogenesis in matrigel plugs through RUNX3 expression recovery.

## DISCUSSION

Mature miRNAs are 21 to 23 nucleotide noncoding RNA molecules that can downregulate the expression of various genes by translational repression [4]. This occurs when complementary sequences are present in the 3′-UTR of the target mRNAs or by directing mRNA degradation [4]. miRNAs can be expressed in a tissue-specific manner and are considered to play important roles in cell proliferation, apoptosis, and differentiation during mammalian development [21]. Moreover, recent studies showed a relationship between miRNA expression patterns and the development of cancer [22] as well as downregulation of specific cancer-related genes [23].

We can suggest that the potential mechanism of miR-130a and miR-495 to target RUNX3 is inhibition of mRNA translation into protein rather than mRNA degradation as they did not changed RUNX3 mRNA expression level. The results showed that miR-130a and miR-495 expression was increased in SNU5 and SNU484 cells under hypoxic conditions (Figure 3). Inhibition of miR-130a and miR-495 upregulated RUNX3 protein expression (Figure 2). miR-130a and miR-495 downregulate RUNX3 protein expression in gastric cancer cells. *Runx3* targeted deletion in mouse lung resulted in lung adenomas and abrogated the cellular defense mechanism against oncogenic activation, suggesting that *Runx3* plays critical roles in normal differentiation and suppression of tumor initiation [14]. In fact, our results showed that anchorage-independent colony formation and cell proliferation were significantly increased (Figure 5C and 5D). The inhibition of RUNX3 target genes, Bim and p21, cell cycle inhibitors, is a one of the mechanisms for the suppression of proliferation.

RUNX3 tumor suppressor function includes an anti-angiogenesis function. RUNX3 destabilizes HIF-1α by recruiting proline hydroxylases (PHDs) to the oxygen degradation domain to increase HIF-1α hydroxylation and proteosomal degradation [24]. These reports suggest that miR-130a and miR-495 play a role in cell cycle progression and angiogenesis in the early stage of tumorigenesis via the inhibition of RUNX3 expression.

Additionally, miR-130a targets ATG2B and Dicer1 to inhibit autophagy and trigger the killing of chronic lymphocytic leukemia cells. miR-130a modulates cell survival programs by regulating autophagic flux. In addition, it is suggested that a relationship between miR-130a and Dicer1 is a regulatory feedback loop that mediates CLL cell survival [25]. miR-495 is upregulated by E12/E47 in breast cancer stem cells and promotes oncogenesis and hypoxia resistance via downregulation of E-cadherin and REDD1 [26]. Various miRNAs targeting RUNX3 have recently been reported in cancer and diseased cells. miR-130a is upregulated in cisplatin-treated hepatocellular carcinoma cells to increase drug resistance [27], but downregulated in diabetic endothelial progenitor cells by targeting RUNX3 [28]. In gastric cancer, miR-106a targeting of RUNX3 is involved in multidrug resistance [29]. miR-106b targets RUNX3 in laryngeal carcinoma cells [30] and miR-532-5p targets RUNX3 expression in cutaneous melanoma. miR-532-5p expression is upregulated in melanoma cells and metastatic melanoma tumors, and anti-miR-532-5p oligomers upregulate RUNX3 expression [31], suggesting that several different miRNAs targeting RUNX3 have specific functions in different types of cancer.

In this study, we identified miR-130a and miR-495 as potential oncomiR candidates capable of targeting RUNX3, decreasing apoptosis, and increasing cell proliferation in gastric cancer cells. Furthermore, they enhanced cancer cell migration and tumor angiogenesis. These results indicated that therapeutics inhibiting miR-130a and miR-495 in SNU5 and SNU484 gastric cancer cells may reduce cell proliferation, migration, and angiogenesis in the early phase of gastric tumorigenesis via RUNX3 expression recovery.

## MATERIALS AND METHODS

### Plasmids

The 3′-UTR of RUNX3 (310 bp), containing the putative miRNA binding sequence, was amplified using PCR from complementary synthesized from total RNA. The used primers, containing the XbaI restriction site, were as follows: forward, 5′-GCTCTAGAGCGGAAGCACGAGGAAAGGAAG-3′ and reverse, 5′-GCTCTAGAGCTCCTTCCACACATCTC AGAG-3′. The PCR product was cloned into the XbaI restriction site downstream of the open reading frame of luciferase in pGL3 control vector to generate the RUNX3 3′-UTR reporter.

### Cell culture and hypoxic conditions

Human gastric cancer cells, SNU5, SNU16, SNU484, MKN1, and MKN45 (American Type Culture Collection, Manassas, VA, USA), were maintained in RPMI-1640 with 10% fetal bovine serum (HyClone, Logan, UT, USA) under normoxic (21% O_2_) conditions at 37°C. For the hypoxic conditions, hypoxic chambers (Thermo Scientific, Waltham, MA, USA and Astec, Fukuoka, Japan) were used to maintain low oxygen tension (1% O_2_, 5% CO_2_, and balanced with N_2_) for 24 h.

### MicroRNA target prediction

Bioinformatic prediction of target genes and miRNA binding sites was performed using three different programs: Target Scan (http://targetscan.org/), miRanda (http://www.microrna.org/microrna/home.do), and Sanger miRbase Target (http://www.mirbase.org/). Only common targets were considered for experimental analysis.

### Microarray analysis

Total RNA from MKN1 cells under hypoxia was collected using TRIzol Reagent (Invitrogen, Carlsbad, CA, USA). GenoSensor human miRNA array was used to compare miRNA expression in gastric cancer cells under normoxic and hypoxic conditions. Total RNA was labeled with biotin and hybridized to the GenoSensor human miRNA array according to the MAUI system. The acquired images and signal intensity of miRNA spots were analyzed with GeneSpring GX (Agilent, CA USA). (Gene Expression Omnibus, file number: GSE 56870).

### Transfection of miRNAs

For overexpression or inhibition of miRNAs, transfection of miRNA mimics (miR-130a, #MSY0000425; miR-495, MSY0002817; miR-330-3p, MSY0000751, Qiagen, Valencia, CA, USA) or miRNA inhibitors (miR-130a inhibitor, MIN0000425; miR-495 inhibitor, MIN0002817, Qiagen) was performed using the HiPerFect transfection reagent (Qiagen), according to the manufacturer's protocol.

### RT-PCR and real-time RT-PCR

First strand cDNA was synthesized from total RNA by using MMLV reverse transcriptase (Promega, Madison, WI, USA). The conditions for semiquantitative PCR were 35 cycles of denaturation (94°C/30 s), annealing (50°C/40 s), extension (72°C/40 s), and final extension (72°C/10 min). The primers used for *RUNX3* were forward, 5′-GAGTTTCACCCTGACCATCACTGTG-3′, reverse, 5′-GCCCATCACTGGTCTT GAAGGTTGT-3′; and for β-actin, forward, 5′-GACTACCTCATGAAGATC-3′, reverse, 5′-GATCCACATCTGCTGGAA-3′. Real-time RT–PCR was performed in a 7500 Real-time PCR system (Applied Biosystems, Carlsbad, CA, USA) with SYBR premix Ex Taq (Takara Bio Inc., Otsu. Japan). Real-time RT–PCR was carried out with specific primers for miScript miR-130a, miR-495 primer assay kit (Qiagen) and β-actin. The PCR program consisted of an initial denaturation step at 95°C for 10 s, followed by 40 cycles of 95°C for 5 s and 60°C for 1 min. The expression level of each specific gene was normalized against the expression of β-actin in the same master reaction. The primers for miRNA-130a were #MS00003444 (Qiagen); for miRNA-495 were #MS00004347 (Qiagen); and for β-actin forward, 5′-GGATGTCCACGTCACACTTC-3′, and reverse, 5′-CACTCTTCCAGCCTTCCTTC-3′. Each experiment was performed in triplicate, and the average was calculated.

### Luciferase assay

For luciferase assays, HEK293 cells were plated into 35-mm dishes 1 day before transfection. Forty-eight hours post-transfection, cell lysates were prepared using reporter lysis buffer (Promega). Luciferase activity was analyzed using a luciferase assay kit (Promega) with a luminometer (Turner Designs, Sunnyvale, CA, USA). β-galactosidase was also analyzed with the enzyme assay kit for the determination of transfection efficiency (Promega). Three independent experiments were performed and assayed in triplicate. The relative luciferase activity was calculated as relative light units (RLU/β-galactosidase).

### Western blot analysis

Proteins were separated by SDS-PAGE and transferred to a nitrocellulose membrane (Whatman, Maidstone, England). The membrane was blocked with 5% non-fat skim milk in Tris-buffered saline containing 0.1% Tween-20 for 1 h at room temperature. The membrane was then incubated with the primary antibody at 4°C overnight, followed by incubation with horseradish peroxidase-conjugated antibody mouse or rabbit immunoglobulins at room temperature for 1 h, and was developed by the West Pico Chemiluminescent Substrate (PIERCE, Woburn, MA, USA).

### Bromodeoxyuridine (BrdU) incorporation assay

SNU484 cells were seeded in 96-well plates and transfected with miR-130a and miR-495 mimics for indicated time period. At the end of treatment, the cells were labeled with BrdU for 2 hours, and incorporated BrdU was quantified using a BrdU Cell Proliferation Assay Kit (Rhoche, Roche Diagnostics, Indianapolis, IN) according to the manufacturer's instructions.

### Colony forming assay

Soft agar colony formation assays were performed using the CytoSelect 96-well Cell Transformation Assay kit (Cell Biolabs, San Diego, CA). Ten days after seeding, the numbers and morphologies of colonies were determined using an inverted phase-contrast microscope (Olympus, Japan). The number of colonies were counted after staining with 0.05% crystal violet for 1 h.

### Matrigel plug assay

Conditioned medium from SNU484 cells transfected with miR-130a and miR-495 inhibitors (50 nM) were mixed with 200 μl matrigel (BD Biosciences) and inoculated subcutaneously into C57Bl/6J mice. After 7 days, the matrigel plugs were removed and photographed. The concentration of hemoglobin in the matrigel plugs was measured using the Drabkin reagent (Sigma, St. Louis, MO, USA) for quantification of blood vessel formation.

### FACS analysis

Cells were plated at a density of 5 × 10^5^ in 60-mm dishes and incubated in DMEM supplemented with 1% fetal bovine serum (HyClone) for 12 h to synchronize the stage of cell cycle. Collected cells were incubated with annexin V and propidium iodide (PI) in 1× annexin V binding buffer for 30 min at 4°C in the dark and then analyzed on BD FACSAria flow cytometer (BD San Diego, CA, USA). Unstained and untreated cells were used as control.

### Statistical analysis

ANOVA was performed to assess the significance of differences among the experimental groups. The level of significance was set at *p* < 0.01 or *p* < 0.05. Results are represented as the mean ± standard deviation.
